# A Distillation Approach to Transformer-Based Medical Image Classification with Limited Data

**DOI:** 10.3390/diagnostics15070929

**Published:** 2025-04-04

**Authors:** Aynur Sevinc, Murat Ucan, Buket Kaya

**Affiliations:** 1 Department of Computer Technologies, Silvan Vocational School, Dicle University, Diyarbakir 21640, Turkey; aynur.sevinc@dicle.edu.tr; 2 Department of Computer Technologies, Vocational School of Technical Sciences, Dicle University, Diyarbakir 21200, Turkey; murat.ucan@dicle.edu.tr; 3 Department of Electronics and Automation, Firat University, Elazig 23119, Turkey

**Keywords:** BeiT, classification, DeiT, distillation, transformers

## Abstract

**Background/Objectives**: Although transformer-based deep learning architectures are preferred in many hybrid architectures due to their flexibility, they generally perform poorly on image classification tasks with small datasets. An important improvement in performance when transformer architectures work with limited data is the use of distillation techniques. The impact of distillation techniques on classification accuracy in transformer-based models has not yet been extensively investigated. **Methods**: This study investigates the impact of distillation techniques on the classification performance of transformer-based deep learning architectures trained on limited data. We use transformer-based models ViTx32 and ViTx16 without distillation and DeiT and BeiT with distillation. A four-class dataset of brain MRI images is used for training and testing. **Results**: Our experiments show that the DeiT and BeiT architectures with distillation achieve performance gains of 2.2% and 1%, respectively, compared to ViTx16. A more detailed analysis shows that the distillation techniques improve the detection of non-patient individuals by about 4%. Our study also includes a detailed analysis of the training times for each architecture. **Conclusions**: The results of the experiments show that using distillation techniques in transformer-based deep learning models can significantly improve classification accuracy when working with limited data. Based on these findings, we recommend the use of transformer-based models with distillation, especially in medical applications and other areas where flexible models are developed with limited data.

## 1. Introduction

The use of transformer architectures in image processing is an important field of study in recent years. The Convolutional Neural Network (CNN), which is used in traditional deep learning architectures, has been replaced by transformer-based architectures that have achieved high success in natural language processing in recent research. However, transformer architectures need to be trained with a large number of images in order to achieve high success. Especially in the field of medical imaging, it is difficult to find datasets containing a large number of data. In addition, epidemics such as SARS [1], COVID-19 [2] and MPox [3] have shown that deep learning-based disease diagnosis processes should not be tolerant to dataset collection processes. It has been observed that the prolonged dataset collection process is an important variable that delays the use of deep learning architectures during epidemics [4,5]. Researchers are working on transformer-based architectures that can achieve high success with datasets containing a small number of images.

The vision transformer model was one of the first to use the transformer architecture in solving computer vision problems and one of the most important architectures in this field. ViT architecture has achieved high success in challenging tasks such as classification and segmentation. Singh et al. worked with ViT architecture on chest X-ray images containing 5863 images in total [6]. They worked on a dataset with two classes, pneumonia and normal. With the advantage of a high number of data and a low number of classes, they classified pneumonia with 97.61% accuracy. Another study using the vision transformer architecture on medical images was conducted by Xin et al. [7]. The HAM10000 dataset, which contains 10,015 skin cancer images in total, was used to classify skin cancer images. The researchers achieved 94.3% success in classifying skin cancer images with vision transformer architecture. Another study on vision transformers and Alzheimer’s disease was conducted by Shin et al. [8]. The researchers worked on a total of 716 images from three classes in the FBB images dataset. They performed training and testing separately with vision transformer and VGG19 architectures. They achieved 66.67% success with VGG19 architecture, which is a relatively old architecture in the literature, and 56.67% success with vision transformer architecture. This study shows that vision transformer architecture has many disadvantages in architectures with low numbers of images.

Research should continue in order for transformer-based architectures to achieve high success in datasets with a low number of images. As can be seen in the reviewed studies, vision transformer architecture is an important architecture with high success. However, this model achieves the promised high success in datasets containing a large number of images.

The data-efficient image transformer (DeiT) model is a transformer-based architecture using a distillation token. This model is an improved version of the model with data distillation tokens. Although the DeiT model is a new deep learning model, it has been used in many fields in the literature. Ferdous et al. trained a dataset of brain MRI images with DeiT architecture [9]. They achieved 93.69% accuracy on a dataset containing 2040 images in total. There are other architectures that focus on improving the performance of transformer-based architectures by using distillation methods. The BeiT architecture uses information distillation methods and masked image patches. Rajesh et al. tried to detect Parkinson’s disease using BeiT architecture and achieved 90% success after training [10]. BeiT architecture has been used in classification tasks not only in medical images but also in ECG signals. Utkars Jain et al. worked on detecting abnormal cardiac rhythm using ECG signals and BeiT architecture [11]. Another interesting study using BeiT architecture was performed by Folle et al. on hand finger images [12]. The researchers named their work DeepNAPSI, in which they performed classification based on BeiT architecture and achieved 88% success in micro-averaging. Studies in this field have focused on the training and results of the data rather than focusing on the reasons for the choice of architecture.

As observed from the reviewed studies in the literature, although the potential of transformer-based architectures in medical image analysis is increasing rapidly, the effectiveness of the methods in brain MR images has not yet been demonstrated comparatively. In particular, the effect of distillation methods on the performance of disease diagnosis in the analysis of medical images has not been clearly investigated.

This study aims to be one of the first studies to evaluate the success of transformer-based architectures in disease diagnosis from medical images and examines whether the use of distillation tokens improves the performance of brain MRI image classification. ViT, DeiT and BeiT models are trained and the results are analyzed in detail. The effect of the use of distillation tokens on performance improvement is analyzed with respect to multiple aspects. The main contributions of the study to the literature are presented below.

- The use of transformer-based models in brain MRI images was investigated and the success of these architectures in classifying brain MRI images was evaluated.
- The effect of the use of distillation methods on classification performance in the field of brain MRI image analysis was investigated.
- The contribution of the Masked Image Modeling (MIM) technique to the classification success of transformer architectures and its effect on the computational load was investigated.
- Which transformer-based architectures should be used and how they should be used in areas such as medical imaging where large datasets cannot be obtained was investigated.

## 2. Materials and Methods

This section describes the datasets utilized in the study and the preprocessing steps applied to the datasets. Then, detailed information about the proposed CNN architecture is given. We also discuss the reasons for choosing this method and the post-processing steps for training the model. The first dataset is the base dataset of our study. The second dataset is utilized while measuring the performance of the developed model when trained on a different dataset. Detailed information about the datasets is presented below.

### 2.1. Dataset and Implementation Details

**Figure 1:**
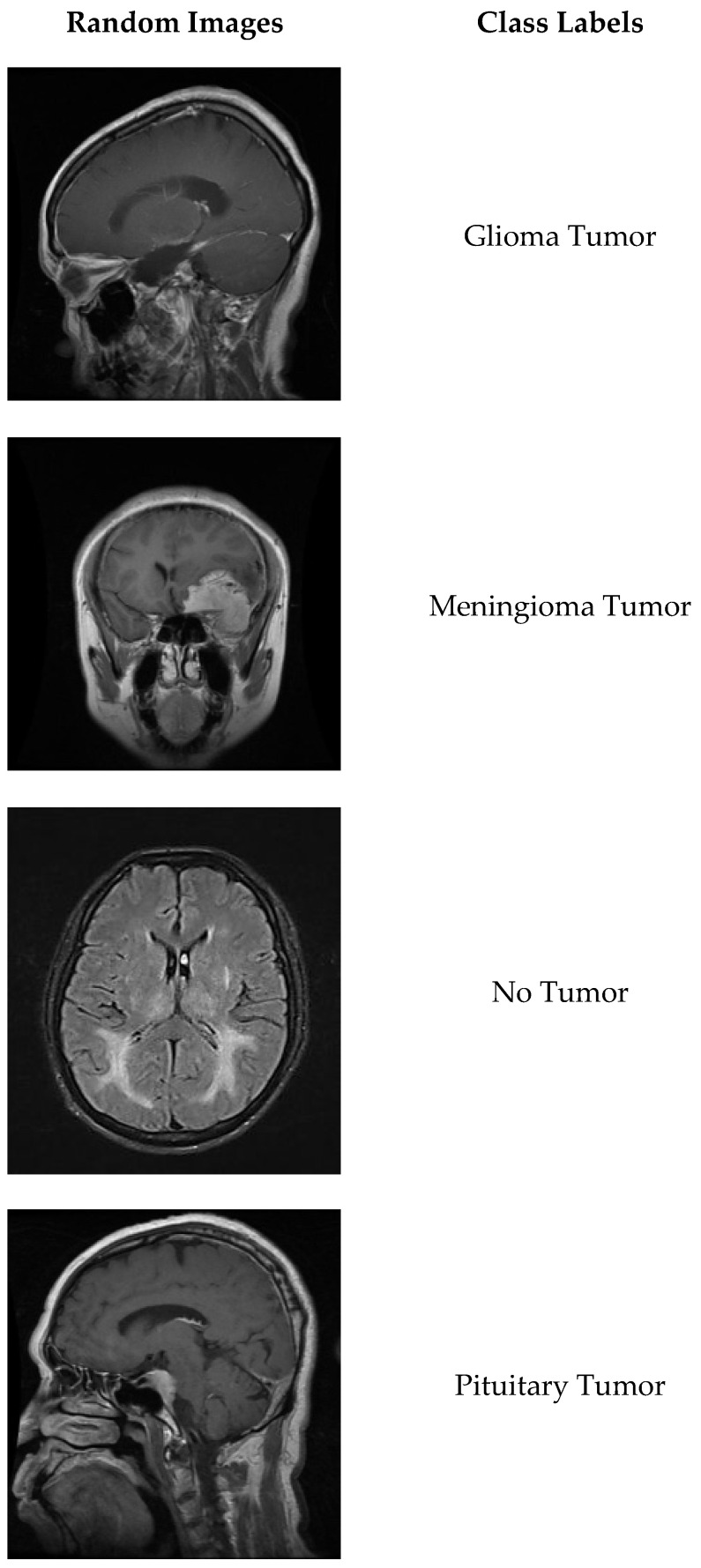
Randomly selected images and class labels from the Brain Tumor Classification (MRI) dataset.

The Brain Tumor Classification dataset, which is an open access dataset containing brain MRI images, was used in the training and testing phases of the study. There are 3264 brain MRI images in total in the dataset, 2870 of which are divided into subsets for training and 394 for tests. The dataset seeks a solution to a multi-class classification problem. The dataset contains images belonging to 4 different types of brain tumors: lioma tumor, meningioma tumor, no tumor and pituitary tumor. Randomly selected images from the dataset are given in Figure 1.

The sample distribution of brain tumor classes used in the study is given in Table 1. The dataset used in the study contains 926, 937, 500 and 901 brain MRI images from glioma tumor, meningioma tumor, no tumor and pituitary tumor classes, respectively. When the dataset used in the study is considered on the basis of disease classes, it can be seen that it has a balanced distribution.

The dataset we used in our study consists of high-quality two-dimensional T1-weighted contrast-enhanced magnetic resonance imaging (MRI) images. The MRI images were examined by expert radiologists and divided into glioma tumor, meningioma tumor, no tumor and pituitary tumor classes, with images belonging to each class in a separate subfolder. Despite detailed literature searches, no demographic information was found for the dataset. However, the fact that the images were provided from different anatomical planes such as axial, coronal and sagittal allowed the deep learning architectures to have a broader perspective on tumor detection and classification processes. The researchers did not share information about the different magnetic field strengths and brands of MRI devices, so no normalization method was applied to remove the magnetic field effect on the images. The MRI images used as inputs to the transformer-based deep learning architectures were resized to fixed pixel sizes to provide a standard input. The purpose of the resizing step Is to ensure a standardized training process when Images are given as inputs to the different deep learning architectures used in the study. For the division of the images in the dataset into training and test subsets, the default division of the publicly available dataset was used. Table 1 shows the training and test subsets of the dataset divided by disease classes. Great care has been taken to ensure that the training and test subsets do not overlap. Any image used in the training phase of the architectures was never used in the testing phase. In this way, patient-independent evaluation of the architectures was ensured and the risk of overfitting was minimized.

This dataset was chosen in order to evaluate the success of transformer architectures in the use of a low number of datasets. The reason for choosing a visual dataset in the medical field is that it is difficult to access medical images in the real world. Many diseases that adversely affect human life and can cause death can be diagnosed using medical images. However, the biggest disadvantage of transformer-based deep learning architectures is that they can achieve high success rates with a large number of training data. This study examines whether this disadvantage of transformer architectures can be solved with solutions such as distillation tokens. In addition, epidemics such as SARS [1], COVID-19 [2] and Mpox [3] have shown that deep learning-based disease diagnosis processes should not be tolerant to dataset collection processes. For these reasons, it is important to achieve high success with transformer architectures based on a low number of datasets.

The MRI Images used In our study were first skull-stripped to remove regions other than the cranial structures. At this stage, the model is focused only on the relevant brain tissues, and thus noise-induced errors are reduced. In the next stage, the light–dark imbalances in the magnetic resonance images were corrected with the bias field correction method. This step enabled the model to extract better features when working on images obtained from different sources. In the slice thickness normalization step, images with different slice thicknesses were brought to similar resolution and detail levels. This step also helped the model to perform more consistently when trained with images from various data sources. The aim of implementing these preprocessing steps is to achieve a more reliable and higher diagnostic success in clinical applications. Before the dataset was given as an input to the deep learning models to be used in the study, it was subjected to some simple preprocessing to provide a more efficient and standardized input. The images were first rescaled to 384 × 384. This processing step ensures that images of different sizes are brought to a standard scale and applied to the model.

### 2.2. Vision Transformer (ViT)

Transformers have been successfully used in deep learning applications such as natural language processing for many years. Feature extraction from images by utilizing the capabilities of transformer architectures is a new field of study today. The vision transformer (ViT) deep learning architecture is a pioneering work in the field that proposes to utilize the capabilities of transformers in image processing [13].

ViT architecture has been used in many fields such as medical images, aviation and agriculture. The architecture, which can be used in image classification and feature extraction from images, can achieve superior success compared to convolutional neural network (CNN)-based models [14]. ViT architecture is more flexible than CNN architectures in understanding changing image sizes and evaluating them globally [15]. Another important advantage of the architecture is its ability to capture the relationships between remote regions of the image. In ViT architecture, images are processed by dividing into predetermined patches such as 16 and 32. Although a filter in CNN architectures traveling over the image and extracting features is good at capturing the relationships between features in close locations, it is an unsuccessful application in determining distant relationships. In ViT architecture, the separation of the image into patches eliminates this disadvantage in CNN architectures and facilitates the capture of distant relationships. The ability to work with irregular data is another superior aspect of the ViT architecture.

It Is a well-known fact that the ViT architecture must work with large amounts of data in order to fully realize its significant advantages. Working with small and unbalanced datasets can negatively affect the success of the architecture. In addition, the architecture uses a large amount of memory due to the attention mechanism. High memory usage makes it difficult to train the dataset with limited resources.

**Figure 2:**
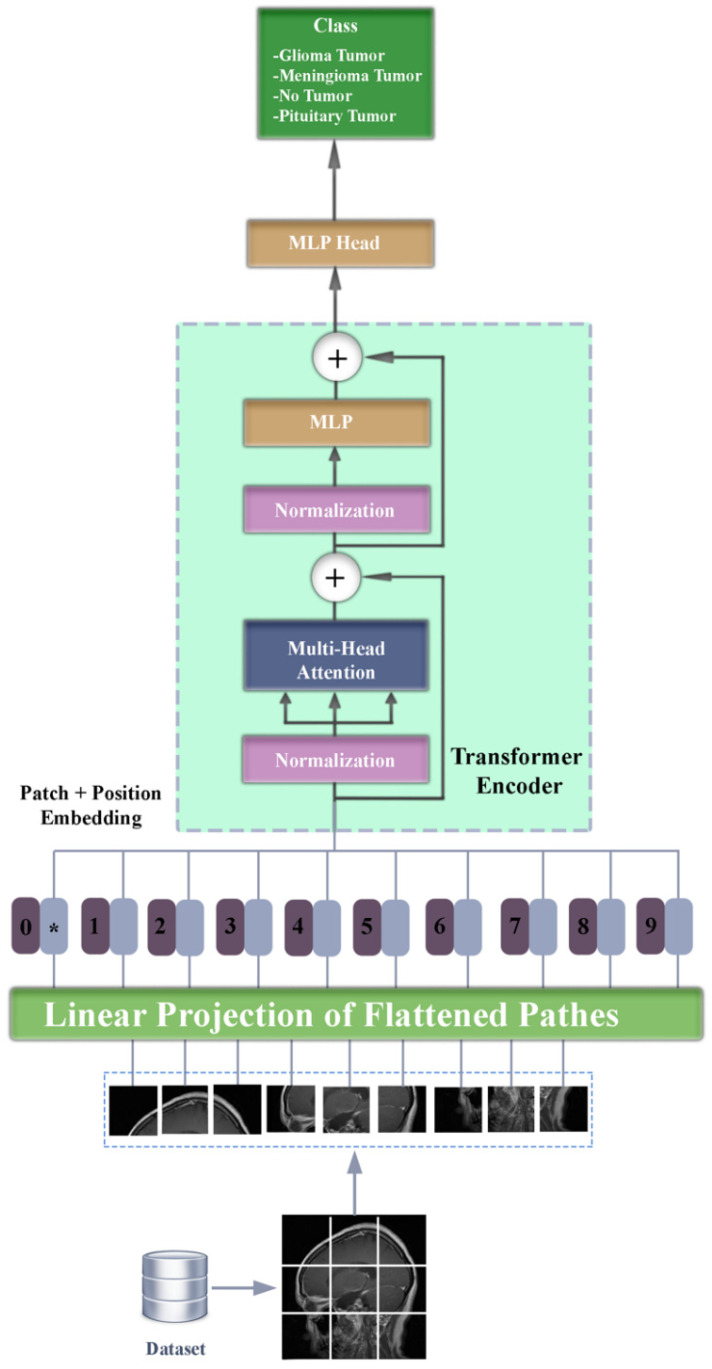
Vision transformer (ViT) model diagram.

Unlike the filter entanglement process in traditional convolutional neural networks, the ViT architecture processes images using an attention mechanism. In the first step of the architecture, the image is divided into equal sized patches. Patches are the basic processing units of the model. Then each patch is transformed into feature vectors in the linear projection layer. In the next step, position vectors are added to each patch to preserve its spatial information. Then the patches are passed through the self-attention layer, respectively. Self-attention calculates the relationship of patches with other patches and long-distance dependencies. By applying the self-attention layer multiple times, features can be learned better by performing multiple calculations. Feature vectors are finalized by processing the outputs of the self-attention layer by the feed-forward network. In the last stage, the representations obtained with the decoder added to the architecture are used to predict the class labels. The block diagram of the described vision transformer architecture is given in Figure 2.

### 2.3. Distilled Data-Efficient Image Transformer

The distilled data-efficient image transformer (DeiT) is a transformer-based deep learning architecture that focuses on achieving high performance on datasets with a low number of images [16]. As with the vision transformer architecture, DeiT aims to utilize the capabilities of the transformer architecture used in natural language processing for image processing. DeiT is a derivative of the ViT architecture enriched with a distillation token [17]. DeiT architecture is an architecture that can achieve high success in areas such as medical imaging, autonomous vehicles and security systems with limited data.

The main disadvantage of transformer-based architectures is that they require large datasets during training. DeiT architecture reduces the high data requirement by using information distillation [18]. The distillation used in the DeiT architecture works as information distillation. Knowledge distillation is based on a real-life teacher–student model. The larger model, the teacher model, transfers information to the smaller model, the student model. A convolutional neural network (CNN) is used in the teacher model, which is a large model, which enables the architecture to learn with smaller data. These advantages indirectly eliminate the training costs and the need for storage capacity during the training of the architecture [19].

**Figure 3:**
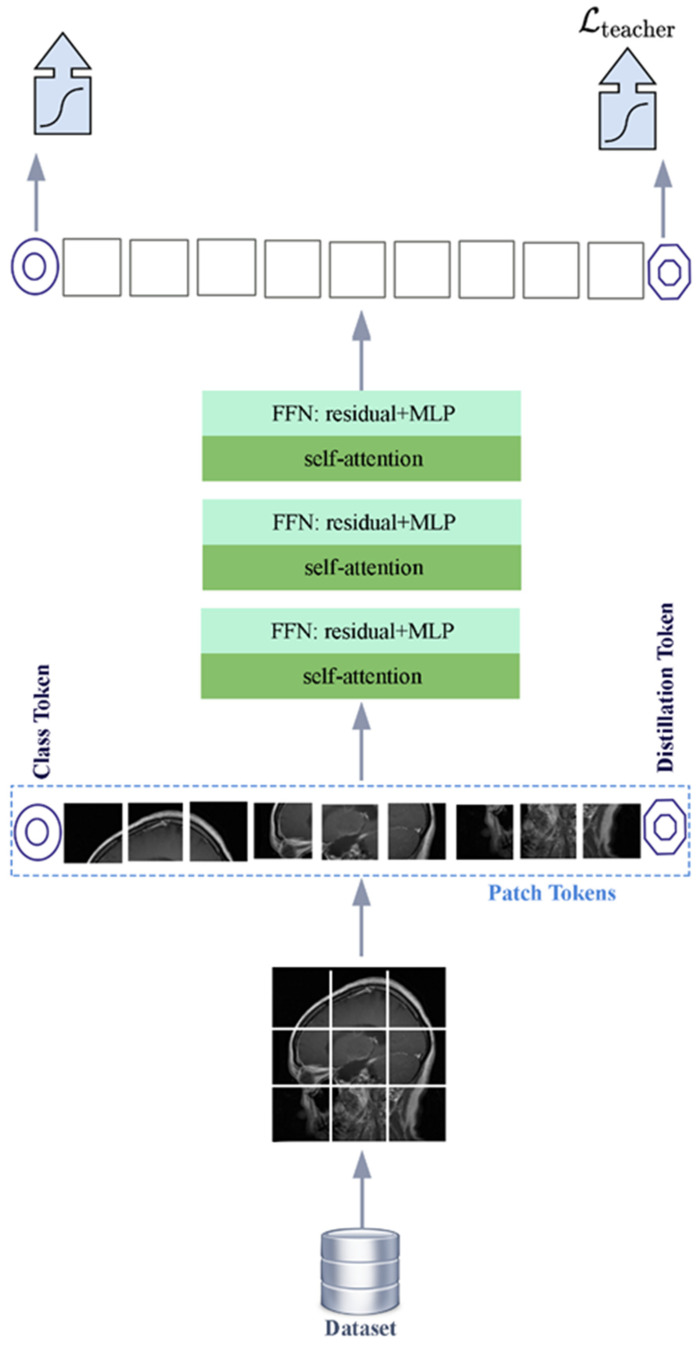
Distilled data-efficient image transformer (DeiT) architecture model diagram.

DeiT architecture starts with the fragmentation of the image into small sub-sections called patches. Distillation tokens and location tags are added to the 16 x16 patches. The patches are then transformed into an embedded representation which is a suitable input to the transformer. As in the ViT architecture, global relations are learned by the architecture with the help of the attention mechanism and feed-forward. The encoder stack consists of three iterative self-attention and FNN layers. Finally, the class labels of the image are obtained with the classification layer. A general representation of the DeiT architecture is given in Figure 3.

Knowledge distillation during training in deep learning architectures has been proposed by Hilton et al. [20]. Knowledge distillation is the process of compressing the knowledge of a small model, called the student, by imitating a large model, called the teacher. There are different versions of knowledge distillation called soft and hard. The main difference between knowledge distillation methods is how the knowledge is transferred to the student model [21]. In soft distillation, the teacher model outputs a statistical distribution. In hard distillation, the output of the model consists of a single label [22].

Since DeiT is an architecture that aims to achieve high success with low amounts of data, a hard information distillation method is used in the architecture. A mathematical representation of the hard information distillation used in the DeiT architecture is given in Formula (1).

(1) $$L_{\mathrm{global}\;}^{\mathrm{HardDistill}}=\frac{1}{2}L_{\mathrm{CE}}\left(\psi \left(Z_{s}\right),y\right)+\frac{1}{2}L_{\mathrm{CE}}\left(\psi \left(Z_{s}\right),y_{t}\right)$$

In the above formula, ψ = Softmax, $L_{\mathrm{CE}}$ = cross-entropy loss, $Z_{s}$ = logits of the student, *y* = ground truth and $y_{t}$= teacher’s predicted labels, respectively. The hard distillation formula basically consists of two parts. The first part of the formula can be expressed as the cross-entropy loss between the model’s predictions and the true labels. This component is a measure of the mismatch between the model predictions and the true class labels. The second part of the formula is the cross-entropy loss between the predictions of the student model and the labels produced by the teacher model. This metric is basically used to bring the results produced by the student model closer to the results produced by the teacher model. The coefficient ½ on both sides of the formula is added so that the student model is equally affected by both the real labels and the guidance of the teacher model during the training process.

The structure of the teacher model plays an important role in the knowledge distillation process in the DeiT deep learning architecture. Thanks to the multi-layered structure of the teacher model, visual representations can be learned in a more powerful way. The teacher model transfers the features acquired during the training process to the student model. The features supported with distillation tokens and location information are transferred to the student model. The distillation tokens and location information added to the transformer-based classification model are shown in Figure 3. This transfer method is an optimization mechanism enabling the student model to attain more accuracy with fewer data requirements. The DeiT deep learning architecture employs a rigorous distillation technique for information transfer. This approach conveys the output of the tutor model to the student model through hard labels. Hard labels utilize precise match data and are optimized to ensure that the output of the student model aligns directly with the predictions of the tutor model. In data-limited fields like medical imaging, the hard distillation method is favored for its stability and strong generalization capabilities. Due to these advantages, the DeiT architecture can attain superior outcomes even when trained on limited datasets.

### 2.4. BERT Pre-Training of Image Transformers (BeiT)

BERT pre-training of image transformers (BeiT) is a transformer-based deep learning architecture used in computer vision applications such as image classification, segmentation and feature extraction from images [23]. The architecture, which enables the use of the competencies of the BERT architecture, which has achieved very successful results in the field of natural language processing of images, was developed by Microsoft Research. BeiT differs from ViT architecture in terms of the pre-training strategy it uses. The Masked Image Modeling (MIM) technique, which is similar to the Masked Language Modeling (MLM) technique frequently used in natural language processing, is an important innovation that distinguishes the architecture from other transformer-based architectures. In the MIM technique, randomly selected patches of the images are masked and then these masked parts are used by the model in predictions.

BeiT architecture can pre-train using unlabeled data. This capability makes the architecture more successful in training and testing processes with fewer labeled data. Being a flexible architecture, BeiT can be used in many challenging tasks such as classification, segmentation, object detection and feature extraction from images. Since it is a transformer-based architecture, it may require high computational power and more training time. Since the MIM technique adds extra data preprocessing steps, it brings extra computational load to the architecture compared to other transformer-based architectures [10].

In BeiT architecture, the model’s learning speed is improved and its performance is improved by using the knowledge distillation token method. In the distillation process, knowledge is transferred from the more powerful teacher model to the student model. The special knowledge distillation method used in the BeiT model optimizes the similarities between the token-based outputs of the teacher model and the token-based inputs of the student model. In this way, the student model has gained a faster and higher performance structure.

BeiT architecture starts with the division of images into 16 × 16 segments. At this stage, a 224 × 224 image is divided into 196 parts in total. Thanks to the fragmentation, operations are carried out on smaller parts of the image called patches. Position placements are added to the patches in the next step. The relationship between patches is learned by using multi-head attention and self-attention in the encoder-decoder mechanism. Masking is applied to some random patches of the image. This means that attempts to estimate the masked parts of the image are made.

**Figure 4:**
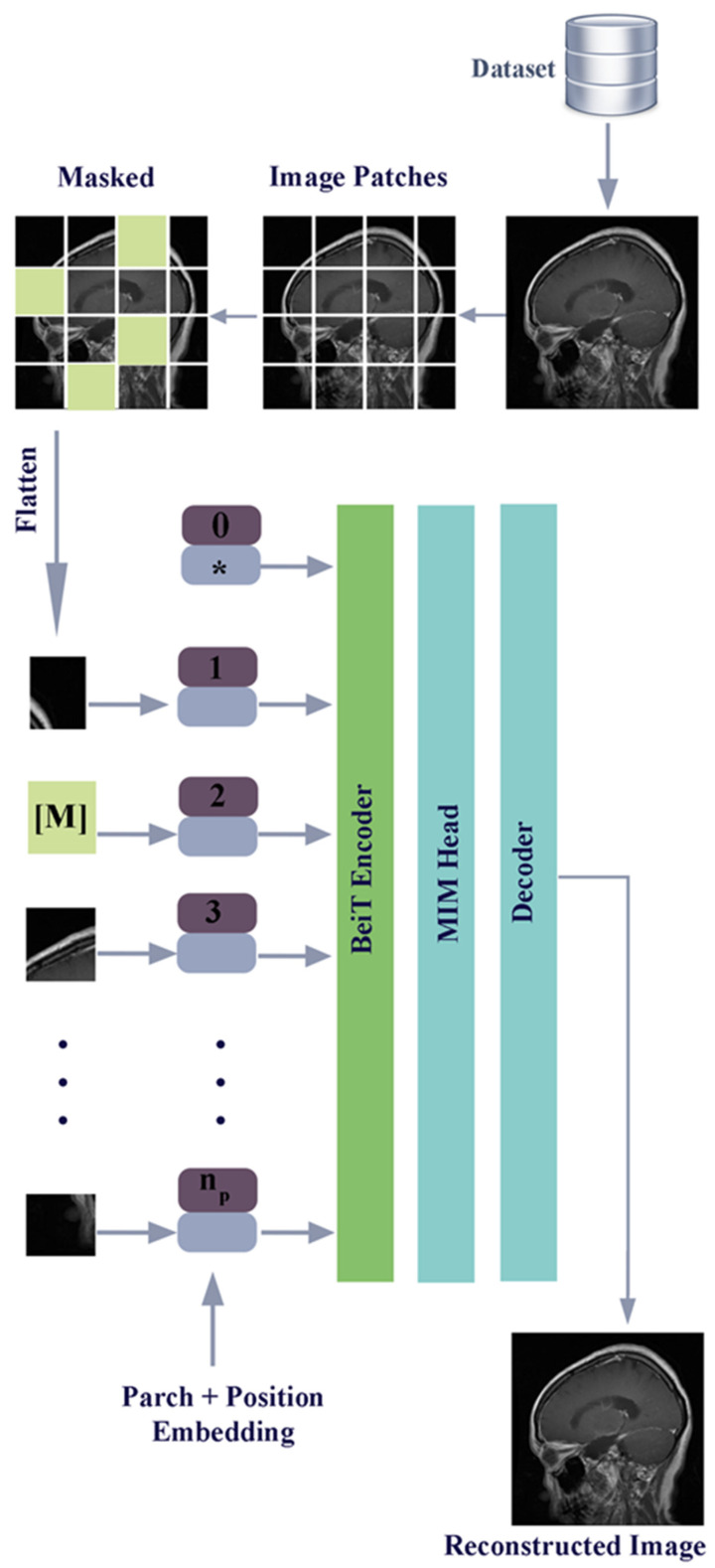
BERT pre-training of image transformers (BeiT) architecture model diagram.

What is important in the MIM technique is the effective use of information from other patches to correctly estimate the masked areas. Bringing the architectural masked parts back into the image is called reconstruction. The VQ-VAE technique is used in the reconstruction phase. In the VQ-VAE technique, predictions are expressed in numerical codes. The pre-training process in which the VQ-VAE technique is used is also called the teacher model. The teacher model transfers information to the student model by learning from the masked patches. Attempts to predict which codes will correspond to the masked parts in the model are made. This is the stage in which information distillation is used. The BeiT model has a 2-stage training process. In the first stage, the model is trained with masked images, the masked parts are learned and the retrieval of the masked parts is learned. In the second stage, the previously learned information is used to fine-tune the classification task. A diagram of the BeiT model with the described stages is given in Figure 4.

### 2.5. Evaluation Metrics

In deep learning architectures, the parameters that enable the performance of the model to be measured and the effectiveness of the model to be tested are called evaluation metrics. Evaluation metrics allow us to understand how well a model performs [24]. One of the evaluation metrics used in deep learning architectures is accuracy. Accuracy is calculated as the ratio of correct predictions to total predictions. This feature allows us to directly compare the classification performance of deep learning architectures and compare them in a measurable way. There are also metrics such as precision, recall and F1 score. Model performance is evaluated by analyzing different metrics [25]. These metrics are calculated using the confusion matrix. The confusion matrix is an important form of representation in which test data are separated according to prediction and actual labels using class labels [26]. Rather than an evaluation metric, the confusion matrix is a representation of the results of the test data using class labels.

## 3. Results

In order to examine the effect of distillation token usage on the success of transformer-based deep learning architectures, a training and test environment was prepared using Python programming language version 3.10.13. P100 GPU was used in the training, validation and testing processes. A P100 GPU was chosen from the Kaggle platform during the training phase, and the experimental setup was set up. The Kaggle platform, a Google developer platform that enables researchers to run Python codes and also provides substantial GPU support, was utilized as the development environment for the faster and more dependable execution of deep learning codes. A total of 100 epoch training cycles were repeated and 32 batch sizes were used. An AdamW optimization algorithm and 5 × 10^−5^ learning rate were preferred. The same parameters were used in all transformer-based deep learning architectures. The hyperparameters used in the studies are given in Table 2 below.

In our study, when training transformer-based deep learning models with limited data, we used weights that were pre-trained with ImageNet as initial weights. This method is preferred because the models trained on ImageNet have high generalization and representation capability and the training process can be used efficiently. This method also helps to shorten the training time by increasing the initial performance of the model with small and specialized datasets and reduces the risk of overfitting, which is an important problem for deep learning architectures. The transfer learning technique also supports the adaptation of deep learning architectures to training data with limited data on medical images, which is the focus of our study. In this context, ImageNet weights were used in our study due to the advantages of transformer-based architectures that converge faster and allow them to work with limited data.

**Figure 5:**
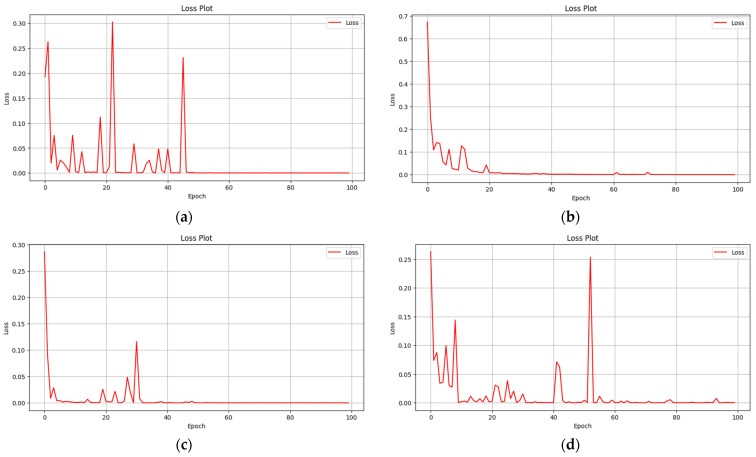
CrossEntropyLoss graphs of transformer-based image classification models: (**a**) ViTx16, (**b**) ViTx32, (**c**) DeiT and (**d**) BeiT.

In our research, we chose CrossEntropyLoss as the loss function due to its widespread use and efficient performance in classification problems. This loss function encourages the model to assign high probabilities to the correct classes by increasing its sensitivity to incorrect predictions. For class imbalance, which is common in medical imaging data, there are other loss functions such as Focal Loss. However, this function gives more weight to minority classes, increasing the sensitivity of the model to these classes. However, since the distribution between the four classes in our dataset was not overly imbalanced, CrossEntropyLoss provided adequate performance. Focal Loss has the disadvantage of hyperparameter settings that are complex, making optimization difficult for small datasets. CrossEntropyLoss, which is a low computational cost and stable option, was found suitable for our study and used in the training process. In deep learning architectures, one of the important parameters by which the success of the architectures is measured is the model loss graph. The most appropriate function to be used for classification problems is analysis of the CrossEntropyLoss graph. CrossEntropyLoss supports the optimization process by measuring the difference between the model outputs and the correct class labels. In the optimization process, CrossEntropyLoss values are used to update the weights of the model. In multi-class classification problems, CrossEntropyLoss focuses on maximizing the correct probabilities of a class while minimizing the probabilities of other incorrect classes. Figure 5 shows the CrossEntropyLoss graphs of the architectures used in the study.

One of the most important representations of classification success in deep learning architectures is the confusion matrix. Rather than being a classification measurement unit, the confusion matrix provides an infrastructure for the calculation of evaluation metrics used in classification. Many evaluation metrics such as accuracy, precision and F-1 score are calculated using this matrix. The confusion matrix has a two-class representation and is also used in multi-class classification problems, such as those in this study. Table 3 shows the classification labels of ViT architecture, one of the transformer architectures without distillation tokens. In the table, classes 0, 1, 2 and 3 represent glioma tumor, meningioma tumor, no tumour and pituitary tumour, respectively.

Vision transformer architecture is the first architecture to utilize the capabilities of transformers in deep learning and no distillation tokens were used. When the classification results obtained in Table 3 are analyzed, it can be seen that non-patient individuals are detected completely correctly with the ViTx32 model. The classification results of transformer-based architectures using the knowledge-based distillation method are also given in Table 4. At this stage, it should be noted that all four architectures were run on exactly the same training and test data. In this way, any possible advantage or disadvantage arising from the random distribution of the datasets is prevented.

The effect of the use of information distillation tokens on classification success was demonstrated by the simulations. DeiT and BeiT architectures were able to detect meningioma tumor and no tumor classes almost without error. Only the DeiT architecture diagnosed a pituitary tumor in one image that should have been labeled as a meningioma tumor. With the exception of this case, the predictions made by both architectures for the two classes are completely correct.

## 4. Discussion

The study was conducted to observe the effect of knowledge-based distillation methods on classification performance in transformer-based architectures. Within the scope of the study, different deep learning architectures with and without distillation were trained and test results were obtained. In this section, quantitative analysis, time performance analysis and parameter efficiency analysis will be carried out in order to examine the superiority of the architectures in more detail.

### 4.1. Quantitative Analysis

It is important to demonstrate the performance of deep learning architectures with measurable parameters in order to be able to compare architectures. Researchers use many metrics, such as accuracy, precision and recall, to show that their deep learning architectures are successful or unsuccessful. In order to obtain these metrics, the test part of the dataset is used. The predictions made in terms of the test data are compared with the real classes and then metrics are calculated with certain mathematical formulas.

Within the scope of the study, two different ViT architectures without distillation techniques and BeiT and DeiT architectures using different distillation techniques were trained and the results obtained were compared with quantitative values. ViTx16, ViTx32, DeiT and BeiT architectures were selected for training and testing processes to compare the effect of distillation techniques on classification success. The effectiveness of the architectures that use distillation techniques and work on transformer-based images has been observed in this study. Table 5 shows a comparison of deep learning methods that use transformer-based distillation techniques and deep learning methods that do not use transformer-based distillation techniques. Classes 0, 1, 2 and 3 in the table represent glioma tumor, meningioma tumor, no tumor and pituitary tumor classes, respectively.

As is well known, it is crucial in classification studies in the field of health to draw clearer lines between sick and non-sick individuals. In other words, a deep learning-based autonomous disease diagnosis application should be able to clearly distinguish between a sick individual and a diseased individual. Diagnosing a non-sick individual with a disease may lead to incorrect treatment and other undesirable problems in the future. Within the scope of the current study, we can assess performance with respect to this situation by examining the classification results for class 2, the no tumor class. In the accuracy evaluation metric of non-patient individuals, 9086%, 8934%, 9315% and 9315% successful classification were obtained in the ViTx32, ViTx16, DeiT and BeiT architectures, respectively. In this context, it is observed that the use of distillation techniques in non-patient individuals provides approximately a 3% increase in disease diagnosis success. In terms of the recall evaluation metric, it was observed that an increase of approximately 5% compared to the ViTx32 architecture and of approximately 9% compared to the ViTx16 architecture was achieved by distillation techniques. For class 1, the meningioma tumor class, there was also an increase in classification success. The use of distillation techniques provided an increase of approximately 1.7% for meningioma tumor detection.

Weighted average accuracy (WAcc) is a frequently used evaluation metric for analyzing unbalanced datasets in deep learning and machine learning algorithms. The use of WAcc is recommended when solving the problem of unrealistic accuracy calculations due to the fact that certain classes have a large number of images and some classes have fewer images.

WAcc is calculated by weighting the accuracy of each class according to the number of samples belonging to that class. In a comparison of architectures using 16 × 16 patches, it was observed that the use of distillation tokens had a positive effect on classification success. Compared to the ViTx16 architecture, the BeiT architecture using the distillation technique had approximately a 1% better classification performance. DeiT architecture, which is another transformer-based architecture using the distillation technique, had an increase in classification performance of approximately 2.2% compared to the ViTx16 architecture.

In order to analyze the reliability of the results obtained in our study, we also conducted a performance comparison with CNN-based architectures in our study. A comparison of the classification performance of popular CNN and transformer-based deep learning architectures in terms of different evaluation metrics is given in Table 6. When the obtained results are analyzed, it is observed that the transformer-based DeiT model achieves the highest classification success with 83.50% accuracy. In addition to accuracy, the DeiT deep learning architecture also achieved 86.99% precision, 82.90% recall and a 82.20% F1 score with respect to other classification metrics. The DenseNet architecture achieved the highest performance among the traditional CNN-based architectures, but it was about 3% lower than the DeiT architecture using distillation tokens. ViT and BeiT architectures also produced competitive results. However, ViT and BeiT architectures, which are transformer-based but do not use distillation tokens, also lagged behind DeiT in performance. The results obtained from the experiments conducted in this study show that the DeiT architecture offers a strong alternative in terms of information distillation and data efficiency.

### 4.2. Time Performance Analysis

Another important parameter for researchers when deciding which deep learning architecture to use in their study is training time. One of the most important advantages of transformer-based systems is flexibility. Researchers produce many solutions that will make life easier by integrating transformer architectures into other systems they have developed. For example, transformer-based architectures can be used as encoders in the feature extraction phase for images, and then medical reports can be written with models such as LSTM, GRU or GPT as decoders. Similarly, transformer architectures are preferred in many areas such as feature extraction, object recognition and segmentation due to their flexible structure. Training time is an important problem, especially for projects that require continuous training or that will be run on mobile devices. Researchers should consider the parameters of time efficiency and classification success together when choosing an architecture. In this context, the time averages for trainings performed in the same development environment for all models with and without the distillation technique were calculated and are given in Table 7. The unit time gain parameter was obtained by calculating the time efficiency of other architectures according to the fastest running architecture. As can be seen from Table 7, the ViTx16 model has 11% more runtime compared to the ViTx32 model. The DeiT model has 7% more runtime compared to the ViTx32 model. The BeiT architecture has a 12% higher runtime compared to the ViTx32 architecture.

### 4.3. Statistical Analysis of the Results

In our study, the statistical significance of the results was analyzed using a one-way ANOVA test with Tukey HSD using different deep learning architectures as independent variables. In research on deep learning architectures, the ANOVA test is a parametric statistical non-significance test used to determine the effect of an independent variable on a dependent variable and evaluate differences by comparing the variance between groups with the variance within groups [27]. If a statistically significant difference is detected as a result of the ANOVA test, Tukey HSD, which also performs statistical analysis, is applied to determine between which groups this difference is found. Tukey HSD analyzes the differences between pairs and reveals whether there is a statistically significant difference between two specific groups with confidence intervals and *p*-values. ANOVA test results for the evaluation metrics obtained in our study are given in Table 8. Statistical pairwise comparison analysis on evaluation metrics is given in Table 9.

As a result of the ANOVA test, an F = 35.3054, *p* < 0.00001 value was obtained, and it was determined that this result was statistically significant at the *p* < 0.05 level. The ANOVA test conducted in the study shows that there are significant differences between the models in terms of different evaluation metrics (SD = 0.0206, df = 3, MS = 0.0069, *p* < 0.05). Subsequently, the Tukey HSD test analyzed the model pairs for which these differences were significant. In particular, significant differences were found in the B1:B2, B2:B3 and B2:B4 comparisons (*p* < 0.00000), and it was determined that the B2 model was statistically superior to the others. On the other hand, no significant differences were observed in the B1:B3 (*p* = 0.84411), B1:B4 (*p* = 0.09742) and B3:B4 (*p* = 0.37954) comparisons. These results confirm that some models outperform others in terms of certain evaluation metrics and that these differences are statistically significant. The statistical analyses used in our study provide a statistically sound basis for model comparisons rather than relying solely on raw accuracy values. The use of statistical significance tests helps to avoid misinterpretations in model selection and provides researchers with information on whether the performances are random or not.

### 4.4. Limitations of the Study and Future Work

This study focuses on the problem of low performance in transformer-based deep learning architectures, which are currently preferred by many researchers for disease diagnosis from medical images, when working with limited data. The contribution of distillation tokens to the classification performance of transformer-based deep learning architectures is investigated in the study. The Brain Tumor Classification dataset and the use of transformer-based architectures constitute the limitations of the study.

Apart from the use of distillation tokens, there are other methods that can be used to improve the successful classification of medical images, such as by using super-resolution. Super-resolution has the potential to improve classification performance by improving image quality in medical image analysis [28]. In this study, we primarily examined the effect of distillation techniques on classification performance in transformer-based deep learning models working with limited data. For this reason, other techniques such as super-resolution were excluded. In future studies, the research team plans to conduct more comprehensive analyses, such as the integration of distillation techniques and super-resolution approaches.

In this study, a technique that will pave the way for increasing the classification success of machine learning and deep learning architectures working on small datasets was studied. The results obtained while working on transformer-based deep learning architectures are intended to guide researchers working on pandemics or rare diseases. Running machine learning and deep learning-based architectures on very large datasets, as well as on small datasets, brings problems such as computational complexity [29]. Another limitation of this study is that it does not work on high-dimensional datasets. The focus of our study was to investigate how to achieve the highest disease diagnosis with low-dimensional datasets for rare diseases or during pandemics.

## 5. Conclusions

The main purpose of this study is to investigate the effect of distillation techniques on the performance of transformer-based architectures on datasets with a low number of datasets. In our study, ViTX32 and ViTX16 architectures, which are transformer-based but do not use distillation techniques, and DeiT and BeiT architectures, which use distillation techniques, were used. A four-class dataset containing brain MRI images was used in the training and testing processes. The results obtained as a result of the training and testing processes using similar parameters and the same development environment were analyzed in detail. In the study, it was observed that the ViTx16 architecture had a classification performance approximately 1% more successful than the BeiT architecture using a distillation technique when distillation token usage was considered with weighted averages. DeiT architecture, which is another transformer-based architecture using a distillation technique, provided an increase of approximately 2.2% in classification success compared to ViTx16 architecture. Looking at the classification results in more detail, it was observed that the use of distillation techniques in the detection of non-patient individuals increased classification performance by approximately 4%. In addition to classification performance, the architectures were also analyzed in terms of time performance. Comparing the architectures using distillation tokens with the ViTx16 architecture, it was observed that the DeiT architecture uses less runtime, while the BeiT architecture consumes only 1% more time.

The main contribution of our study to the literature is its investigation of the effect of the use of distillation techniques in transformer-based architectures on classification success in small datasets. Our study on small datasets will guide those working in the field of medicine, especially in special cases such as epidemics or in other diseases for which it is difficult to create large datasets. Our study has shown that distillation techniques can provide significant classification success when working with limited data. The limitations of our study are related to the application of images as inputs to transformer-based architectures and the dataset containing brain MRI images. Another limitation is that demographic and clinical data were not shared by the researchers who created the dataset used in the study, which makes it difficult to evaluate the generalizability of the developed architectures. In addition, it is unclear whether the magnetic field intensity is homogeneous in all images since it is not shared in the sources of the dataset or in other studies in the literature. These limitations should be taken into account when interpreting the findings of the study. In future studies, hybrid distillation techniques and how distillation techniques can be improved will be studied.

## Figures and Tables

**Table 1:** Distribution of brain tumor class instance numbers of the dataset used in the study.

Class	Train	Test	Total
Glioma Tumor	826	100	926
Meningioma Tumor	822	115	937
No Tumor	395	105	500
Pituitary Tumor	827	74	901

**Table 2:** Hyperparameters used in the deep learning architectures.

Hyperparameter	Value
Batch Size	32
Learning Rate	5 × 10^−5^
Optimizer	AdamW
Train Epochs	100

**Table 3:** Classification results for test images obtained from deep learning architectures based on transformers without knowledge-based distillation.

Predicted Class									
		ViTx32				ViTx16			
		0	1	2	3	0	1	2	3
Actual Class	0	42	31	26	1	31	36	31	2
	1	0	112	3	0	0	114	1	0
	2	0	1	104	0	0	0	105	0
	3	0	6	6	62	0	1	10	63

**Table 4:** Classification results for test images obtained from transformer-based deep learning architectures with knowledge-based distillation.

Predicted Class									
		DeiT				BeiT			
		0	1	2	3	0	1	2	3
Actual Class	0	46	32	21	1	34	42	19	5
	1	0	114	0	1	0	115	0	0
	2	0	0	105	0	0	0	105	0
	3	3	1	6	64	0	1	8	65

**Table 5:** Separate classification results with different evaluation metrics for all classes obtained from the test images and the weighted average accuracy value calculated.

		0			1		2			3			Average
	Accuracy	Precision	Recall	Accuracy	Precision	Recall	Accuracy	Precision	Recall	Accuracy	Precision	Recall	WAcc
ViTx32	0.85	0.42	1.0	0.90	0.97	0.75	0.91	0.99	0.75	0.97	0.84	0.98	0.901
ViTx16	0.82	0.31	1.0	0.90	0.99	0.75	0.89	1.0	0.71	0.97	0.85	0.97	0.892
DeiT	0.85	0.46	0.94	0.91	0.99	0.78	0.93	1.0	0.80	0.97	0.86	0.97	0.914
BeiT	0.83	0.34	1.0	0.90	1.0	0.73	0.93	1.0	0.80	0.96	0.88	0.93	0.900

**Table 6:** Comparison of classification performance of popular CNN and transformer-based deep learning architectures with respect to different evaluation metrics.

Model	Accuracy	Precision	Recall	F1 Score
ResNet50	0.8020	0.8659	0.7895	0.7844
DenseNet121	0.8046	0.8700	0.8008	0.7815
ViTx32	0.8122	0.8697	0.8056	0.7986
ViTx16	0.7944	0.8596	0.7882	0.7676
BeiT	0.8096	0.8630	0.8046	0.7847
DeiT	0.8350	0.8699	0.8290	0.8220
Mean	0.8096	0.8664	0.803	0.7898
Std.Dev.	0.0139	0.0043	0.0148	0.0186

**Table 7:** Time averages for the trainings performed in the same development environment.

Model	Time (s)	Unit Time Gain
ViTx32	6162	100%
ViTx16	6844	111%
DeiT	6602	107%
BeiT	6915	112%

**Table 8:** ANOVA test results for evaluation metrics obtained from test results for deep learning architectures used in experiments.

Source	SS	df	MS
Between-Evaluation Metric	0.0206	3	0.0069
Within-Evaluation Metric	0.0039	20	0.0002
Total	0.0244	23	

**Table 9:** Statistical pairwise comparison analysis of different evaluation metrics.

		HSD_0.05_ = 0.0225	Q_0.05_ = 3.9583
		HSD_0.01_ = 0.0285	Q_0.01_ = 5.0180
B_1_:B_2_	M_1_ = 0.81	0.06	Q = 9.99 (*p* = 0.00000)
	M_2_ = 0.87		
B_1_:B_3_	M_1_ = 0.81	0.01	Q = 1.16 (*p* = 0.84411)
	M_3_ = 0.80		
B_1_:B_4_	M_1_ = 0.81	0.02	Q = 3.48 (*p* = 0.09742)
	M_4_ = 0.79		
B_2_:B_3_	M_2_ = 0.87	0.06	Q = 11.15 (*p* = 0.00000)
	M_3_ = 0.80		
B_2_:B_4_	M_2_ = 0.87	0.08	Q = 13.47 (*p* = 0.00000)
	M_4_ = 0.79		
B_3_:B_4_	M_3_ = 0.80	0.01	Q = 2.32 (*p* = 0.37954)
	M_4_ = 0.79		

## Data Availability

The data supporting the findings of this study are based on a publicly available dataset in the reference: [30].
